# Survival outcome assessment for triple-negative breast cancer: a nomogram analysis based on integrated clinicopathological, sonographic, and mammographic characteristics

**DOI:** 10.1007/s00330-022-08910-4

**Published:** 2022-06-27

**Authors:** Dan-li Sheng, Xi-gang Shen, Zhao-ting Shi, Cai Chang, Jia-wei Li

**Affiliations:** 1 Department of Medical Ultrasound, Fudan University Shanghai Cancer Center, No 270, Dong’an Road, Xuhui District, Shanghai, 200032 China; 2 Department of Oncology, Shanghai Medical College, Fudan University, Shanghai, 200032 China; 3 Department of Radiology, Fudan University Shanghai Cancer Center, Shanghai, 200032 China

**Keywords:** Triple-negative breast neoplasms, Disease-free survival, Ultrasonography, Mammography, Nomograms

## Abstract

**Objective:**

This study aimed to incorporate clinicopathological, sonographic, and mammographic characteristics to construct and validate a nomogram model for predicting disease-free survival (DFS) in patients with triple-negative breast cancer (TNBC).

**Methods:**

Patients diagnosed with TNBC at our institution between 2011 and 2015 were retrospectively evaluated. A nomogram model was generated based on clinicopathological, sonographic, and mammographic variables that were associated with 1-, 3-, and 5-year DFS determined by multivariate logistic regression analysis in the training set. The nomogram model was validated according to the concordance index (C-index) and calibration curves in the validation set.

**Results:**

A total of 636 TNBC patients were enrolled and divided into training cohort (*n* = 446) and validation cohort (*n* = 190). Clinical factors including tumor size > 2 cm, axillary dissection, presence of LVI, and sonographic features such as angular/spiculated margins, posterior acoustic shadows, and presence of suspicious lymph nodes on preoperative US showed a tendency towards worse DFS. The multivariate analysis showed that no adjuvant chemotherapy (HR = 6.7, 95% CI: 2.6, 17.5, *p* < 0.0005), higher axillary tumor burden (HR = 2.7, 95% CI: 1.0, 7.1, *p* = 0.045), and ≥ 3 malignant features on ultrasound (HR = 2.4, CI: 1.1, 5.0, *p* = 0.021) were identified as independent prognostic factors associated with poorer DFS outcomes. In the nomogram, the C-index was 0.693 for the training cohort and 0.694 for the validation cohort. The calibration plots also exhibited excellent consistency between the nomogram-predicted and actual survival probabilities in both the training and validation cohorts.

**Conclusions:**

Clinical variables and sonographic features were correlated with the prognosis of TNBCs. The nomogram model based on three variables including no adjuvant chemotherapy, higher axillary tumor load, and more malignant sonographic features showed good predictive performance for poor survival outcomes of TNBC.

**Key Points:**

• *The absence of adjuvant chemotherapy, heavy axillary tumor load, and malignant-like sonographic features can predict DFS in patients with TNBC.*

• *Mammographic features of TNBC could not predict the survival outcomes of patients with TNBC.*

• *The nomogram integrating clinicopathological and sonographic characteristics is a reliable predictive model for the prognostic outcome of TNBC.*

**Supplementary Information:**

The online version contains supplementary material available at 10.1007/s00330-022-08910-4.

## Introduction

Triple-negative breast cancer (TNBC), a molecular type of breast cancer with negative expression of estrogen receptor (ER), progesterone receptor (PR), and human epidermal growth factor receptor-2 (HER-2), accounts for approximately 15 to 20% of all breast cancer cases [1–3]. Compared with patients with non-TNBC subtypes, those with TNBC have a higher risk of relapse, metastatic disease, and worse survival outcomes [4, 5]. The median survival time of TNBC patients with metastatic disease is only 13–18 months [6, 7]. Owing to its aggressive behavior and unfavorable outcome, effective treatment of TNBC remains a clinical challenge, and thus, efforts to develop predictive models for prognosis are important.

Some clinicopathological factors, including age at diagnosis, positive axillary lymph node status, lymphovascular invasion (LVI), and histological grade, have been observed to be associated with prognosis in TNBC patients [8–11]. Advanced transcriptomic and genomic subtypes have also been shown to influence the treatment outcomes and clinical prognosis of TNBCs. Shao et al proposed a new TNBC subtyping system named the Fudan University Shanghai Cancer Center classification system which includes four molecular subtypes: immunomodulatory, luminal androgen receptor, basal-like immune-suppressed (BLIS), and mesenchymal. Each subtype has a characteristic immune or genomic signature that can be an effective target [12]. In addition, a clinical trial found that combined molecular subtyping and genomic profiling greatly enhanced the therapeutic efficiency in refractory metastatic TNBC [13].

Radiologic imaging, such as magnetic resonance imaging (MRI), mammography (MG), and ultrasound (US), are widely used as preoperative diagnostic techniques. Importantly, imaging characteristics in these modalities were also found to be associated with the survival outcome of TNBC. Among these, MRI features have been extensively investigated owing to their intrinsic advantages with respect to standard scanning procedures and semiquantitative analysis, and the results showed that these features are associated with the prognosis of TNBCs. It has been demonstrated that the absence of preoperative MRI, tumor-stromal ratio, and peritumoral edema on T_2-_ weighted images were correlated with worse prognosis in TNBC patients [14–16].

Meanwhile, compared with MRI, US and MG features are less investigated despite them being more commonly used techniques in preoperative evaluation. Previous findings in the US showed that compared with tumors classified as Breast Imaging and Reporting Data System (BI-RADS) category 4B-5, category 4A tumors have poorer survival outcomes [17]. Another study showed that breast cancers with sonographic features of vertical orientation were more likely to have a higher risk of recurrence [18]. The presence of mammographic features of casting-type calcification and architectural distortions or dense breast tissue have been reported to be associated with an increased risk of recurrence in patients with TNBC [14, 19, 20]. However, data on predicting the prognosis of TNBCs based on integrated clinicopathological and imaging features are lacking.

Therefore, the present study aimed to identify clinicopathological, sonographic, and mammographic characteristics predictive of survival outcomes of TNBC and to develop and validate an effective predictive nomogram model for TNBC prognosis.

## Materials and methods

### Study design and population

This retrospective study was approved by the institutional review board of Fudan University Shanghai Cancer Center. The need for written informed consent was waived owing to the retrospective nature of the study.

A total of 876 women diagnosed with TNBC at our institution between January 2011 and December 2015 were enrolled. Among them, we excluded patients with (1) neoadjuvant chemotherapy (*n* = 101); (2) history of surgery for malignant breast tumor (*n* = 31); (3) incomplete or loss of data on clinicopathological characteristics and preoperative US images (*n* = 87); (4) bilateral malignant lesions (*n* = 5); (5) lesions without a mass on US (*n* = 10); and (6) diagnosis of metastatic disease beyond the breast and axilla at presentation (*n = *6). Finally, 636 patients with TNBC were included in the analysis.

### Data collection

Clinicopathological information was obtained from the patient medical records. Data on age, body mass index, menopausal status, surgery type, and postoperative adjuvant treatment were collected. Pathological data included tumor size, histological type, histological grade, axillary lymph node involvement, LVI, HER2 score, and Ki-67 expression level. Histologically, the tumors were categorized as grade I, highly differentiated; grade II, moderately differentiated; and grade III, poorly differentiated. Axillary lymph node involvement was determined by either sentinel lymph node biopsy (SLNB), and/or axillary lymph node dissection (ALND). The axillary tumor burden was classified as negative (no tumor involved lymph nodes), low (1–3 tumors involved lymph nodes), and heavy tumor load (≥ 4 tumors involved lymph nodes) [14, 21]. The Body mass index (BMI) was calculated as weight/hight^2^.

### Variable definition

The status of ER and PR was considered negative if less than 1% of tumor cells had nuclear staining [22]. HER2 negativity was defined HER2 score of 0 or 1+ in immunohistochemistry or as absent HER2 amplification in fluorescence in situ hybridization. TNBC was defined as a simultaneous negative expression of ER, PR, and HER2. Ki-67 expression levels were categorized as high expression and low expression at a cutoff of 40% [23].

Disease-free survival (DFS) was defined as the time from the date of surgery to the date of local-regional recurrence, distant metastasis, or occurrence of contralateral breast cancer. All patients were followed up until December 2020. Those without DFS events were censored at the last follow-up.

### Assessment of sonographic and mammographic images

US images were retrospectively collected from the institutional image archive servers. US was performed using Aixplorer (Supersonic Imaging), Logic E9 (GE Healthcare), XMATRIX and IU22 (Philips Medical Systems), Aplio 500 (Toshiba medical system), and Mylab90 and MylabTwice (Esaote). Each breast tumor mass was assessed with respect to size (maximum diameter), orientation, shape, margin, echo halo, echo pattern, posterior acoustic pattern, and calcification. For patients with multiple lesions, only the largest tumor mass was evaluated. Abnormal axillary lymph nodes on US were defined as those with irregular cortical thickness ≥ 3 mm, longest/shortest axis ratio < 2, or absence of fatty hilum [24, 25]. To avoid inter-observer variations in BI-RADS scores, all breast masses were reevaluated and classified into three subcategories based on the number of malignant features in US images: no malignant sonographic features; 1–2 malignant sonographic features, and ≥ 3 malignant sonographic features [26]. Malignant sonographic features included vertical orientation, irregular shape, uncircumscribed angular and/or spiculated margin, presence of echo halo, posterior acoustic shadow, and presence of calcification [27, 28]. The sonographic features were evaluated by two US physicians, with at least 5 years’ experience in breast imaging, according to the fifth edition of the ACR BIRADS® Atlas.

All mammograms were performed using digital MG units (Selenia Dimensions, HOLOGIC, and Inspiration, SIEMENS). Breast density was classified as non-dense (predominantly fatty or scattered fibroglandular) and dense (heterogeneously or extremely dense). Each lesion was described as a mass, calcification only, architectural distortion, asymmetry, and normal mammographic findings. The shapes and margins of the masses and the morphology and distribution of calcifications were then evaluated. All the breast lesions were also reevaluated and classified into three groups according to the number of malignant features in mammographic images: no malignant mammographic features; 1–2 mammographic malignant features; and ≥ 3 malignant mammographic features. The malignant mammographic features included irregular shape, uncircumscribed margin, calcification morphology (amorphous, coarse heterogeneous, fine pleomorphic, and fine branching), calcification distribution (grouped, segmental, and branching), architectural distortion, and asymmetry [28]. All mammographic features were assessed by two radiologists with at least 5 years’ working experience in MG.

All assessments were performed independently, and all four physicians were blinded to the other assessments.

### Statistical analysis

**Fig. 1:**
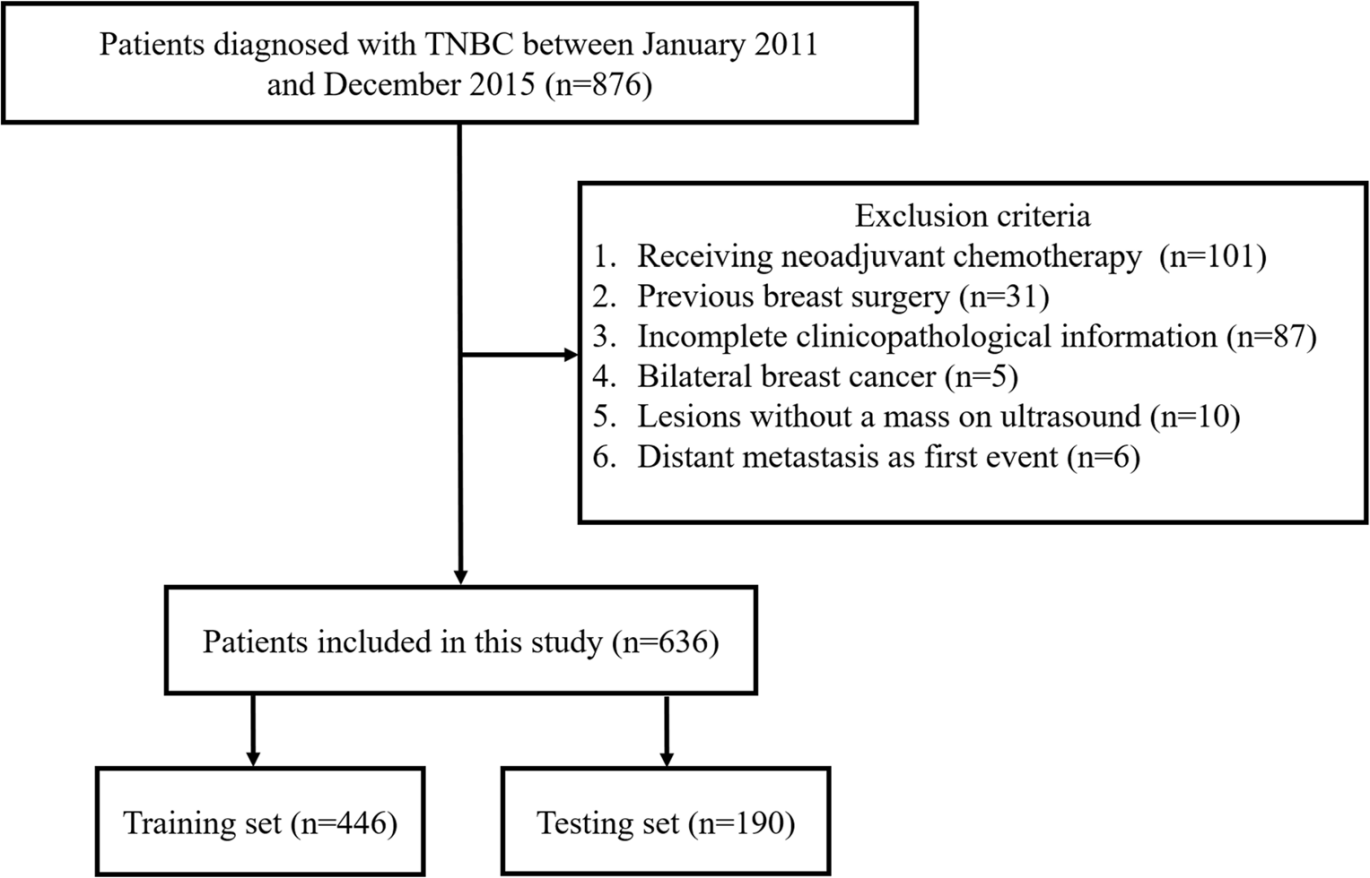
Workflow for patient enrollment in the study

For nomogram construction, the patients were divided into training and validation cohorts in ratio of 7:3 (Fig. 1). The differences in clinicopathologic and imaging characteristics between the training and validation cohorts were evaluated using an independent sample *t*-test or chi-square test. In the training cohort, the Cox proportional hazards regression model was used for univariate and multivariate analyses to identify variables significantly (*p* < 0.05) associated with DFS. Hazard ratios (HRs) and 95% confidence intervals (CIs) for each variable were calculated.

The nomogram for predicting 1-, 3-, and 5- year DFS in patients with TNBC was developed according to the results of the multivariate analysis. The discriminative capability of the established model was evaluated according to the concordance index (C-index), receiver operating characteristic curve (ROC), and area under the ROC curve (AUC). Calibration curves were constructed to compare the predicted DFS with the observed DFS using a bootstrap approach with 1000 resamples. Data were presented as the mean ± standard deviation (SD), median [interquartile range, IQR], or number (%). All statistical analyses were performed using SPSS (version 22.0, SPSS Inc.) and R software (version 4.1.0). A two-tailed *p* value < 0.05 was considered statistically significant.

## Results

### Patient characteristics

A total of 636 patients (*n* = 446 in the training cohort and 190 in the validation cohort) were included in this study (Fig. 1). All patients underwent preoperative US examination, while only 458 patients had mammographic data (*n* = 316 in the training cohort and *n* = 142 in the validation cohort). Data on clinicopathological, sonographic, and mammographic characteristics of the cohorts are summarized in Tables S1-S3 and the recurrence percentages for each feature were provided in Table S4. Except for the types of mass shape (oval/round, irregular, and no mass) in MG (*p* = 0.034), there was no significant difference in other characteristics between the two cohorts (all *p* > 0.05).

The median age of the study population was 52.1 ± 11.3 years, and the median follow-up time was 67.3 [55.1–82.9] months. DFS events occurred in 100 patients. Among these patients, 20 (20.0%), 42 (42.0%), 25 (25.0%), 9 (9.0%), and 4 (4.0%) patients had locally recurrence, distant recurrence, both local-regional and distant recurrence, contralateral breast cancer, and both contralateral breast cancer and distant recurrence, respectively.

### Influencing factors associated with DFS in the training cohort

Univariate survival analysis showed that tumor size > 2 cm (*p* = 0.019), axillary dissection (*p* = 0.005), metastasis in four or more axillary lymph nodes (*p* < 0.0005), presence of LVI (*p* = 0.002), and no adjuvant chemotherapy (*p* < 0.0005) were associated with worse DFS outcomes (Table 1). Sonographic features associated with recurrence were angular and/ or spiculated margins (*p* = 0.018), posterior acoustic shadows (*p* < 0.0005), presence of suspicious lymph nodes on preoperative US (*p* = 0.002), and ≥ 3 malignant features on US (*p* < 0.0005) (Table 2). However, no mammographic features, including breast density (*p* = 0.437), lesion type (*p* = 0.997), calcification (*p* = 0.173), mass shape (*p* = 0.835), mass margin (*p* = 0.813), calcification morphology (*p* = 0.269), calcification distribution (*p* = 0.390), and number of malignant features in MG (*p* = 0.285), were predictive of DFS events (Table 3).

**Table 1:** Univariate analysis of clinicopathological features associated with DFS

Variables	All patients (*n* = 446)	Recurrence (*n* = 70)	No recurrence (*n* = 376)	Hazard ratio	*p* value
Age (year)					0.950
< 55	255 (57.2)	39 (55.7)	216 (57.4)	Reference	
≥ 55	191 (42.8)	31 (44.3)	160 (42.6)	1.02 (0.63, 1.63)	
Menopausal status					0.583
Premenopause	210 (47.1)	30 (42.9)	180 (47.9)	Reference	
Menopause	236 (52.9)	40 (57.1)	196 (52.1)	1.14 (0.71, 1.83)	
Tumor size in pathology (mm)					0.019
≤ 20	191 (42.8)	21 (30.0)	170 (45.2)	Reference	
> 20	255 (57.2)	49 (70.0)	206 (54.8)	1.84 (1.10, 3.07)	
Type of surgery					0.699
Breast-conserving surgery	80 (17.9)	12 (17.1)	68 (18.1)	Reference	
Mastectomy	366 (82.1)	58 (82.9)	307 (81.9)	0.88 (0.47, 1.65)	
Axillary surgery					0.005
SLNB	197 (44.2)	19 (27.1)	178 (47.3)	Reference	
Axillary dissection	249 (55.8)	51 (72.9)	198 (52.7)	2.11 (1.25, 3.58)	
Histological type					0.848
Invasive ductal carcinoma	424 (95.1)	66 (94.3)	358 (95.2)	Reference	
Other invasive carcinoma	22 (4.9)	4 (5.7)	18 (4.8)	1.10 (0.40, 3.03)	
Histological grade					0.617
Grade II	74 (17.3)	13 (19.4)	61 (16.9)	Reference	
Grade III	354 (82.7)	54 (81.6)	300 (83.1)	0.86 (0.47, 1.57)	
Axillary lymph node load					< 0.0005
No positive lymph node	288 (64.6)	34 (48.6)	254 (67.6)	Reference	
1–3 positive lymph nodes	104 (23.3)	15 (21.4)	89 (23.7)	1.26 (0.69, 2.35)	0.454
≥ 4 positive lymph nodes	54 (12.1)	21 (30.0)	33 (8.7)	4.03 (2.34, 6.95)	0.000
Lymphovascular invasion					0.002
Absent	290 (65.0)	34 (48.6)	256 (68.1)	Reference	
Present	156 (35.0)	36 (51.4)	120 (31.9)	2.11 (1.32, 3.38)	
Ki-67					0.096
≤ 40%	148 (33.2)	30 (42.9)	118 (31.4)	Reference	
> 40%	298 (66.8)	40 (57.1)	258 (68.6)	0.67 (0.42, 1.07)	
Adjuvant chemotherapy					0.000
Yes	421 (94.4)	62 (88.6)	359 (95.5)	Reference	
No	10 (2.2)	6 (8.6)	4 (1.1)	5.84 (2.53, 13.59)	0.000
Unknown	15 (3.4)	2 (2.8)	13 (3.4)	0.94 (0.23, 3.84)	0.930
Adjuvant radiotherapy					0.208
Yes	155 (34.7)	30 (42.9)	125 (33.2)	Reference	
No	276 (61.9)	38 (54.3)	238 (63.3)	0.65 (0.40, 1.05)	0.079
Unknown	15 (3.4)	2 (2.8)	13 (3.5)	0.67 (0.16, 2.80)	0.581
BMI					0.877
< 25	337 (75.6)	52 (74.3)	285 (75.8)	Reference	
25–30	96 (21.5)	18 (25.7)	78 (20.7)	1.15 (0.67, 1.97)	0.610
> 30	13 (2.9)	0 (0.0)	13 (3.5)	0.000 (0.000, 6.524E+229)	0.965

**Table 2:** Univariate analysis of sonographic features associated with DFS

Variables	All patients (*n* = 446)	Recurrence (*n* = 70)	No recurrence (*n* = 376)	Hazard ratio	*p* value
Orientation					0.298
Parallel	392 (87.9)	59 (84.3)	333 (88.6)	Reference	
Vertical	54 (12.1)	11 (15.7)	43 (11.4)	1.41 (0.740, 2.68)	
Shape					0.441
Regular	73 (16.4)	10 (14.3)	63 (16.8)	Reference	
Irregular	373 (83.6)	60 (85.7)	313 (83.2)	1.30 (0.67, 2.54)	
Margin					0.002
Circumscribed	60 (13.4)	5 (7.2)	55 (14.6)	Reference	
Angular/spiculated	169 (37.9)	40 (57.1)	129 (34.3)	3.08 (1.22, 7.80)	0.018
Indistinct/microlobular	217 (48.7)	25 (35.7)	192 (51.1)	1.42 (0.54, 3.71)	0.476
Echogenic halo					0.687
Absent	431 (96.6)	67 (95.7)	364 (96.8)	Reference	
Present	15 (3.4)	3 (4.3)	12 (3.2)	1.27 (0.40, 4.03)	
Echo pattern					0.115
Hypoechoic	237 (53.1)	31 (44.3)	206 (54.8)	Reference	
Mixed hypo/iso-echoic	178 (39.9)	35 (50.0)	143 (38.0)	1.65 (1.02, 2.68)	0.042
Mixed hypo/an-echoic	31 (7.0)	4 (5.7)	27 (7.2)	1.06 (0.37, 2.99)	0.919
Posterior acoustic pattern					< 0.0005
Enhancement	171 (38.3)	19 (27.1)	152 (40.4)	Reference	
Shadow	56 (12.6)	21 (30.0)	35 (9.3)	3.64 (1.95, 6.76)	< 0.0005
Mixed change	219 (49.1)	30 (42.9)	189 (50.3)	1.20 (0.68, 2.12)	0.543
Calcification					0.391
Absent	339 (76.0)	50 (71.4)	289 (76.9)	Reference	
Present	107 (24.0)	20 (28.6)	87 (23.1)	1.26 (0.75, 2.11)	
Suspicious lymph nodes in US					0.002
Absent	337 (75.6)	43 (61.4)	294 (78.2)	Reference	
Present	109 (24.4)	27 (38.6)	82 (21.8)	2.16 (1.33, 3.49)	
Number of malignant features					< 0.0005
No malignant feature	49 (11.0)	8 (11.4)	41 (10.9)	1.61 (0.73, 3.53)	0.235
1–2 malignant features	293 (65.7)	28 (40.0)	265 (70.5)	Reference	
3 or more malignant features	104 (23.3)	34 (48.6)	70 (18.6)	3.74 (2.26, 6.16)	< 0.0005

**Table 3:** Univariate analysis of mammographic features associated with DFS

Variables	All patients (*n* = 316)	Recurrence (*n* = 43)	No recurrence (*n* = 273)	Hazard ratio	*p* value
Breast density					0.437
Nondense	100 (31.6)	16 (37.2)	84 (30.8)	Reference	
Dense	216 (68.4)	27 (62.8)	189 (69.2)	0.78 (0.42, 1.45)	
Lesion type					0.997
Mass	227 (71.8)	32 (74.4)	195 (71.4)	Reference	
Calcification only	7 (2.2)	0 (0.0)	7 (2.6)	0 (0, NA)	0.978
Architectural distortion	10 (3.2)	1 (2.3)	9 (3.3)	0.69 (0.09, 5.08)	0.720
Asymmetry	65 (20.6)	9 (21.0)	56 (20.5)	0.99 (0.46, 2.01)	0.909
Normal mammographic findings	7 (2.2)	1 (2.3)	6 (2.2)	0.85 (0.12, 6.24)	0.875
Calcification					0.173
Absent	220 (69.6)	26 (60.5)	194 (71.1)	Reference	
Present	96 (30.4)	17 (39.5)	79 (28.9)	1.15 (0.83, 2.82)	
Mass shape					0.835
Oval/round	57 (18.0)	7 (16.3)	50 (18.3)	1.04 (0.40, 2.68)	0.939
Irregular	170 (53.8)	25 (58.1)	145 (53.1)	1.22 (0.60, 2.48)	0.583
None	89 (28.2)	11 (25.6)	78 (28.6)	Reference	
Mass margin					0.813
Circumscribed	15 (4.7)	0 (0.0)	15 (5.5)	0.000 (0.000, 3.116E+249)	0.968
Noncircumscribed	212 (67.1)	32 (74.4)	180 (65.9)	1.25 (0.63, 2.48)	0.521
None	89 (28.2)	11 (25.6)	78 (28.6)	Reference	
Calcification morphology					0.269
Amorphous	12 (3.8)	4 (9.3)	8 (2.9)	3.52 (1.23, 10.11)	0.019
Coarse heterogeneous	5 (1.6)	0 (0.0)	5 (1.8)	0.000 (0.000, 1.320E+250)	0.971
Fine pleomorphic	53 (16.8)	8 (18.6)	45 (16.5)	1.30 (0.59, 2.87)	0.516
Fine branching	4 (1.3)	1 (2.3)	3 (1.1)	2.94 (0.40, 21.69)	0.291
Benign	22 (6.9)	4 (9.3)	18 (6.6)	1.44 (0.50, 4.13)	0.499
None	220 (69.6)	26 (60.5)	194 (71.1)	Reference	0.019
Calcification distribution					0.390
Regional	7 (2.2)	0 (0.0)	7 (2.6)	0.98 (0.00, NA)	0.977
Grouped	58 (18.4)	9 (20.9)	49 (17.9)	1.37 (0.64, 2.92)	0.416
Segmental	9 (2.8)	3 (7.0)	6 (2.2)	2.90 (0.39, 21.39)	0.297
Branching	4 (1.3)	1 (2.3)	3 (1.1)	3.23 (0.98, 10.70)	0.054
Scattered	18 (5.7)	4 (9.3)	14 (5.1)	1.74 (0.61, 4.98)	0.305
None	220 (69.6)	26 (60.5)	194 (71.1)	Reference	
Number of malignant features					0.285
No malignant features	67 (21.2)	7 (16.3)	60 (22.0)	Reference	
1–2 malignant features	32 (10.1)	2 (4.6)	30 (11.0)	0.59 (0.12, 2.82)	0.506
3 or more malignant features	217 (68.7)	34 (79.1)	183 (67.0)	1.53 (0.68, 3.44)	0.311

The variables significantly associated with DFS events in the univariate analysis were further included in the multivariate Cox regression analysis. Among them, no adjuvant chemotherapy (HR = 6.7, 95% CI: 2.6, 17.5, *p* < 0.0005), ≥ 4 axillary lymph node metastases (HR = 2.7, 95% CI: 1.0, 7.1, *p* = 0.045), and ≥ 3 malignant features on US (HR = 2.4, CI: 1.1, 5.0, *p* = 0.021) were identified as independent variables associated with poorer DFS outcomes (Table 4).

**Table 4:** Multivariate analysis of features associated with DFS

Variables	All patients (*n* = 446)	Recurrence (*n* = 70)	No Recurrence (*n* = 376)	Hazard ratio	*p* value
Tumor size (mm)					0.116
≤ 20	191 (42.8)	21 (30.0)	170 (45.2)	Reference	
> 20	255 (57.2)	49 (70.0)	206 (54.8)	1.58 (0.89, 2.79)	
Axillary surgery					0.784
SLNB	197 (44.2)	19 (27.1)	178 (47.3)	Reference	
Axillary dissection	249 (55.8)	51 (72.9)	198 (52.7)	0.91 (0.45, 1.82)	
Axillary load					0.014
No positive lymph nodes	288 (64.6)	34 (48.6)	254 (67.6)	Reference	
1–3 positive lymph nodes	104 (23.3)	15 (21.4)	89 (23.7)	0.96 (0.40, 2.33)	0.934
4 or more positive lymph nodes	54 (12.1)	21 (30.0)	33 (8.7)	2.70 (1.02, 7.13)	0.045
Lymphovascular invasion					0.978
Absent	290 (65.0)	34 (48.6)	256 (68.1)	Reference	
Present	156 (35.0)	36 (51.4)	120 (31.9)	0.99 (0.45, 2.19)	
Adjuvant chemotherapy					0.001
Yes	10 (2.2)	6 (8.6)	4 (1.1)	Reference	
No	421 (94.4)	62 (88.6)	359 (95.5)	6.70 (2.56, 17.53)	0.000
Unknown	15 (3.4)	2 (2.8)	13 (3.4)	1.41 (0.34, 5.96)	0.637
Margin					0.534
Circumscribed	60 (13.4)	5 (7.2)	55 (14.6)	Reference	
Spiculated/angular	169 (37.9)	40 (57.1)	129 (34.3)	1.91 (0.58, 6.31)	0.289
Indistinct/microlobular	217 (48.7)	25 (35.7)	192 (51.1)	1.45 (0.50, 4.18)	0.492
Posterior acoustic pattern					0.724
Enhancement	56 (12.6)	21 (30.0)	35 (9.3)	Reference	
Shadow	171 (38.3)	19 (27.1)	152 (40.4)	1.38 (0.60, 3.14)	0.447
Mixed change	219 (49.1)	30 (42.9)	189 (50.3)	1.23 (0.65, 2.34)	0.526
Suspicious lymph nodes in ultrasound					0.389
Absent	337 (75.6)	43 (61.4)	294 (78.2)	Reference	
Present	109 (24.4)	27 (38.6)	82 (21.8)	1.31 (0.71, 2.39)	
Number of malignant features in ultrasound					0.031
No malignant feature	49 (11.0)	8 (11.4)	41 (10.9)	1.96 (0.79, 4.84)	0.145
1–2 malignant features	293 (65.7)	28 (40.0)	265 (70.5)	Reference	
3 or more malignant features	104 (23.3)	34 (48.6)	70 (18.6)	2.40 (1.15, 5.04)	0.021

### Nomogram construction and validation

Based on the three prognostic factors identified in the multivariate Cox analysis, a nomogram model was established to predict 1-, 3-, and 5-year DFS.

**Fig. 2:**
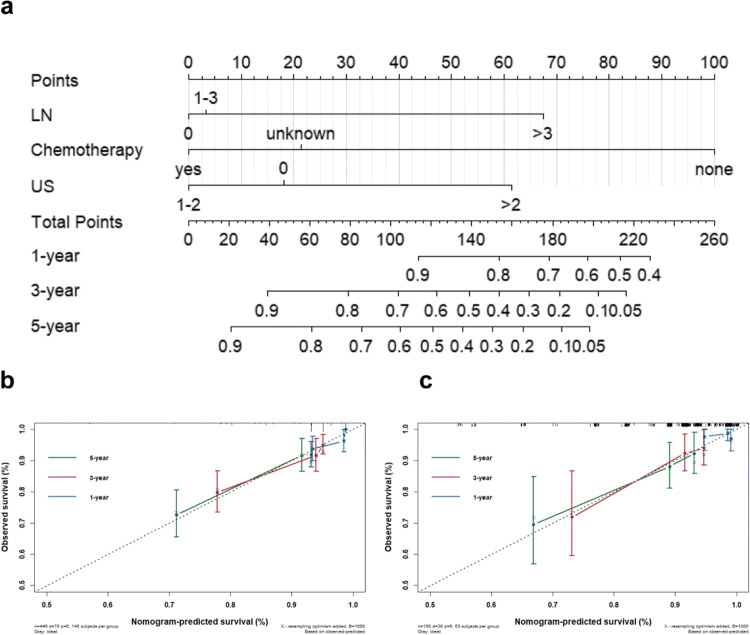
Constructed nomogram and its calibration plots. (**a**) Nomogram of predicting 1-, 3-, and 5-year DFS for TNBC patients. LN: lymph node. Calibration plots of predictions for the 1-, 3-, and 5- year DFS in the training set (**b**) and the validation set (**c**)

As shown in Fig. 2a, the absence of adjuvant chemotherapy had the highest impact, followed by the number of axillary lymph node metastases and the number of malignant sonographic features. The corresponding survival probability of each patient was obtained by summing the specific points from these prognostic variables.

With respect to the predictive performance of the nomogram, the C-index for predicting DFS was 0.693 for the training cohort and 0.694 for the validation cohort, indicating good discrimination capability. The calibration plots for the 1-, 3-, and 5-year DFS exhibited excellent consistency between the nomogram-predicted and actual survival probabilities in the training and validation cohorts (Fig. 2b). Subsequently, the ROC curves for 1-, 3-, and 5-year DFS in both the training and validation cohorts were drawn. The AUCs for predicting 1-, 3-, and 5-year DFS were 0.786, 0.706, and 0.691, respectively, in the training cohort and were 0.488, 0.725, and 0.691, respectively in the validation cohort (Figure S1). A comparison between the nomogram and the American Joint Committee on Cancer (AJCC) tumor, nodes and metastases staging system (eighth edition) showed that the nomogram had a higher AUC with respect to predicting DFS in the training cohort (Figure S2).

**Fig. 3:**
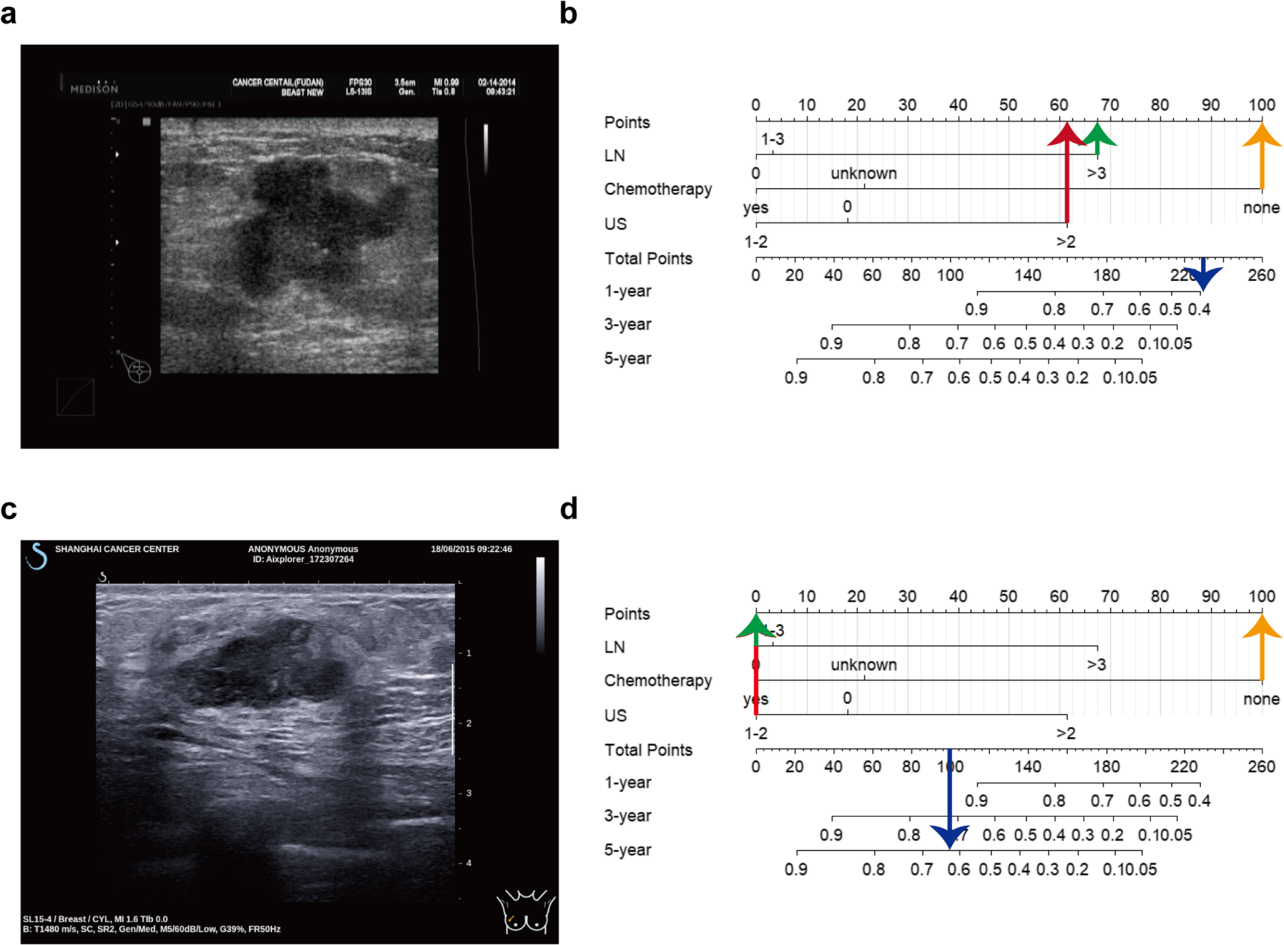
Examples of the nomogram model. **a** Case 1: a representative case with three malignant sonographic features (irregular shape, angular margin, and presence of calcification). **b** The nomogram shows the total points of case 1: absence of adjuvant chemotherapy (100 points, yellow arrow) + 4 four metastatic axillary lymph nodes (67.5 points, green arrow) + three malignant sonographic features (61.5 points, red arrow) = 229. The corresponding probability of 1-year DFS in case 1 calculated by the nomogram model was less than 40 % (blue arrow). **c** Case 2: a representative case with one malignant sonographic feature (irregular shape). **d** The nomogram shows the total points of case 2: absence of adjuvant chemotherapy (100 points, yellow arrow) + no metastatic axillary lymph node (0 points, green arrow) + one malignant sonographic features (0 poinst, red arrow) = 100. The corresponding probability of 5-year DFS in case 2 calculated by the nomogram model was about 62 % (blue arrow)

Representative examples of estimating the survival probabilities of specific patients are shown in Fig. 3. The patient in Fig. 3a did not receive adjuvant chemotherapy and had four metastatic axillary lymph nodes and three malignant sonographic features (irregular shape, uncircumscribed angular margin, and presence of calcification). The probability of 1-year DFS calculated using the nomogram model was ≤ 40 % (Fig. 3b). The patient was diagnosed with bone metastasis 6.6 months postoperatively. Similarly, patient 2 in Fig. 3c, who had one malignant sonographic feature (irregular shape) without chemotherapy, and no lymph node metastasis, had a 62 % probability of 5-year DFS (Fig. 3d). No recurrence event was observed in the patient 5 years postoperatively.

## Discussion

TNBC is well known to have poor outcomes owing to its aggressive biological characteristics and lack of targeted therapy [4]. Thus, there have been efforts to predict and improve the prognosis of TNBCs. This study found that the absence of adjuvant postoperative chemotherapy, higher tumor load in axillary lymph nodes, and malignant-like sonographic appearances are risk factors for 1-, 3-, and 5-year DFS in TNBC patients. The nomogram model established based on these three prognostic variables was confirmed to be a reliable prognostic model for discrimination and calibration analyses. As far as we know, we firstly incorporated clinicopathological, sonographic, and mammographic characteristics to predict the survival of TNBC patients.

As shown in the nomogram, no adjuvant chemotherapy was strongly associated with poor DFS. TNBC does not benefit from endocrine or targeted molecular therapies because it lacks drug-targeted receptors. Anthracycline-/taxane-based chemotherapy remains the mainstay of systemic treatment for TNBC, greatly improving its outcomes [29, 30]. Neoadjuvant chemotherapy also plays an increasingly important role in TNBC treatment, particularly in patients with locally advanced or unresectable cancers. One study found no significant difference in survival between adjuvant and neoadjuvant chemotherapy [31]. However, although adjuvant chemotherapy had a beneficial impact on survival outcomes, some patients with TNBC, such as the elderly or those with comorbidities, are not eligible for adjuvant chemotherapy. Among these patients, those with high levels of stromal tumor-infiltrating lymphocytes in the early stage had excellent survival outcomes; thus, a subset of TNBC patients could be spare adjuvant chemotherapy [32, 33].

Consistent with previous studies [14, 34–36], we found that TNBC patients with more metastatic axillary lymph nodes had a worse prognosis. This is expected because the number of positive lymph nodes determines the pathological stage of breast cancer and is important predictor of survival outcomes [37]. The surgery type of axilla and suspicious lymph nodes in US were significantly correlated with the DFS in the univariate survival analysis while they were excluded in the final nomogram model. This might be explained by the collinearity between these two variables and the number of metastatic axillary lymph nodes. Similarly, tumor size and presence of LVI were also not included in the nomogram model. The role of tumor size in predicting TNBC prognosis was still debated [38]. It has been reported that smaller tumors might have aggressive biology and unfavorable prognosis [39]. However, it was also reported that larger tumors (sizes > 2 cm or 5 cm) were the risk factor for worse survival [40]. Our study was consistent with those of a population-based study of 1601 breast TNBC patients that concluded that tumor size was not a determinate factor for the prognosis of TNBCs [3]. LVI refers to the invasion of tumor emboli into lymphatic spaces or blood vessels in the peritumoral area [41]. Although the mechanism of LVI remains unclear, it has been identified as an independent prognostic factor for patients with high risk, including the TNBC subtype [42]. In this study, the LVI also was not identified as a determinative variable in the prediction model considering the potential collinearity with the number of positive lymph nodes in the axilla.

Our study found that patients whose tumors had more than three malignant sonographic features might have inferior RFS than those with no or 1–2 malignant features. This finding was in concordance with previous findings [43]. The angular/spiculated margin and posterior acoustic attenuation are well-known typical sonographic features of malignant breast tumors. The angular/spiculated margin is believed to be associated with the low proliferation rates of malignant cells, which allow enough time to have stromal interactions and induce fibrosis surrounding the invasive edge [44]. The fibrosis as well as the disorganized growth of malignant cells lead to an increase in acoustic impedance that causes posterior acoustic attenuation. These two sonographic features were reported to indicate the aggressive behaviors of TNBC [18]. It also has been reported that tumors with severe fibrosis are less responsive to chemotherapy as the fibrous extracellular matrix hinders the penetration of chemotherapeutic agents [11]. Similarly, Wand et al found that the presence of vertical orientation was correlated with angular margin and posterior acoustic attenuation, which reflect aggressive behaviors and predict worse outcomes in TNBC [18]. Elsawaf et al reported that infiltrative borders in TNBC were associated with the luminal cluster and poor outcomes while pushing border pattern tended to have a basal cluster and good prognosis [45]. These findings support that malignant-like TNBCs have poorer prognosis than those with benign sonographic appearance. Therefore, considering the collinearity with the number of malignant sonographic features, the presence of angular/spiculated margin and posterior acoustic attenuation were not included in the nomogram model.

Interestingly, the positive relationship between the malignant-like sonographic features and the poor prognosis of TNBCs was contrary to our previous finding that TNBCs with benign-like sonographic features have aggressive biological properties [27, 46] and a higher recurrence risk [26]. Some other researches also came to the controversial conclusion that TNBCs with circumscribed margins and posterior acoustic enhancement share more proliferative and aggressive biological properties. Our previous study [26] and the study by Elfgen et al [47] show that although BLIS has a higher probability of presenting with benign-like sonographic features, it tends to have poorer survival outcomes than the other three subtypes of TNBC. While the two studies only identify the relationship between ultrasound characteristics and different subtypes of TNBCs, they did not analyze the association between sonographic features and disease outcomes directly. These contradictory findings suggest the heterogeneity of TNBC with respect to sonographic features, biological properties, and clinical behaviors. We advocate more comprehensive studies incorporating radiomics, proteomics, and genomic information to elucidate the relationship among the subtypes of TNBC, sonographic features, and prognosis. This is being undertaken at our cancer center in collaboration with the departments of radiology, breast surgery, and pathology.

Surprisingly, in contrast with previous studies, no mammographic features influenced the survival of TNBCs. It had been reported that the presence of mammographic features of casting-type calcification and architectural distortions are associated with poorer survival outcomes in patients with TNBC [19, 20]. Bae et al also demonstrated that TNBC patients with high breast density on MG had an increased risk of recurrence [14]. The primary reason for the contrasting findings might be that all lesions enrolled in our study were mass-type lesions on US. This might cause selection bias in MG, which also indicates the limitation of MG for the detection of such breast lesions [47].

Some limitations should be considered when interpreting the results of our study. First, the retrospective study design and lack of genomic data may weaken the reliability of the predictive model. A well-designed prospective study including radiomic, proteinic, and genomic data which is being undertaken at our center would confirm our results. Second, MRI imaging data were not included in the predictive model because of the small number of preoperative breast MRI scans. This will be supplemented in our future studies. Third, the predictive model was established based on infiltrative TNBCs presenting as a mass on US, and the non-mass type lesions were not included.

## Conclusion

Clinical factors including tumor size > 2 cm, axillary dissection, presence of LVI, absence of adjuvant postoperative chemotherapy, heavy tumor load in axillary lymph nodes (≥ 4 positive lymph nodes), and sonographic features such as angular/spiculated margins, posterior acoustic shadows, presence of suspicious lymph nodes on preoperative US, and malignant-like sonographic appearances (≥ 3 malignant US features) were all associated with worse DFS of TNBC. The final nomogram model integrating three variables of adjuvant postoperative chemotherapy, heavy tumor load in axillary lymph nodes, and malignant-like sonographic appearances could serve as an effective and convenient tool to predict survival outcomes for TNBC patients. Future research involving genomic information will be conducted to further verify our findings.

## Supplementary information

ESM 1 (DOCX 526 kb)

## Abbreviations

AJCC: American Joint Committee on Cancer
ALND: Axillary lymph node dissection
AUC: Area under the curve
BI-RADS: Breast Imaging and Reporting Data System
BLIS: Basal-like immune-suppressed
BMI: Body mass index
CI: Confidence interval
C-index: Concordance index
DFS: Disease-free survival
ER: Estrogen receptor
HER2: Human epidermal growth factor receptor-2
HR: Hazard ratio
IQR: Interquartile range
LVI: Lymphovascular invasion
MG: Mammography
MRI: Magnetic resonance imaging
PR: Progesterone receptor
ROC: Receiver operating characteristic curve
SD: Standard deviation
SLNB: Sentinel lymph node biopsy
TNBC: Triple-negative breast cancer
US: Ultrasound

## Declarations

### Guarantor

This study was funded by the National Natural Science Foundation of China (No. 81627804, 81830058, 81901760, and 82102070), and Shanghai Anticancer Association SOAR Project (No. SACA-AX201905).

### Statistics and biometry

One of the authors has significant statistical expertise.

### Study subjects or cohorts overlap

The research team has been working on the association among sonographic features, biological properties, and clinical outcomes of TNBCs since 2018 with a series of publications with different study objectives and designs. There were some inevitable overlaps in the patient cohorts. There were 636 patients in the present study. A cohort of 75 patients in this study were published in the previous study in Scientific Reports in 2018 (reference 27 in the present study) in which the sonographic variety of TNBCs was addressed. A cohort of 81 patients in this study were published in the *Chinese Journal of Ultrasonography* in 2019 (not referred to in the present study) in which the feasibility study of using quantitative sonographic features to correlate with biological properties was evaluated. A cohort of 88 cases were published in Annals of Translational Medicine in 2020 (reference 41 in the present study) in which we found the sonographic appearances of TNBCs are associated with the four molecular subtypes based on mRNA and lncRNA. Sonographic features of TNBCs had the trend to correlate with the survival outcome. Meanwhile, the same cohort of patients was then published in Frontiers in Oncology in 2021 (reference 26 in the present study) in which an integrated predictive model based on sonographic features was established for evaluating the risk of tumor recurrence estimated from the mRNAs and lncRNAs. A cohort of 92 patients were published in European Radiology (reference 43 in the present study) in which the performance to predict tumor biological property using quantitative high-throughput feature analysis was compared with that of two-dimensional sonographic feature assessment.

### Methodology

• Retrospective

• Diagnostic or prognostic study

• Performed at one institution
