# Supplementary material for: Survival outcome assessment for triple-negative breast cancer: a nomogram analysis based on integrated clinicopathological, sonographic, and mammographic characteristics
Source: Eur Radiol. 2022 Jun 27;32(10):6575–87. doi: 10.1007/s00330-022-08910-4 (PMC9474369; doi:10.1007/s00330-022-08910-4)
Supplement: Supplementary file 1 — (DOCX 526 kb) [file 330_2022_8910_MOESM1_ESM.docx]

| **Table S1 Comparison of clinicopathological characteristics in training and validation sets** | | | |
| --- | --- | --- | --- |
| **Variables** | **Training set (n=446)** | **Validation set (n=190)** | **P value** |
| Age (year) | 51.9 ± 11.6 | 52.4 ± 10.6 | 0.585 |
| Follow-up time (month) | 66.9 ± 22.0 | 68.3 ± 19.4 | 0.443 |
| Ki-67 (%) | 57. 2 ± 24.3 | 57.8 ± 24.3 | 0.763 |
| BMI (kg/m^2^) | 23.2 ± 3.0 | 23.4 ± 3.1 | 0.639 |
| Tumor size in pathology (mm) | 25.1 ± 10.3 | 23.8 ± 9.6 | 0.129 |
| Menopausal status |  |  | 0.673 |
| Premenopause | 210 (47.1) | 86 (45.3) |  |
| Menopause | 236 (52.9) | 104 (54.7) |  |
| Type of surgery |  |  | 0.740 |
| Breast-conserving surgery | 80 (17.9) | 32 (16.8) |  |
| Mastectomy | 366 (82.1) | 158 (83.2) |  |
| Axillary surgery |  |  | 0.331 |
| SLNB | 197 (44.2) | 76 (40.0) |  |
| Axillary dissection | 249 (55.8) | 114 (60.0) |  |
| Histologic type |  |  | 0.478 |
| Invasive ductal carcinoma | 424 (95.1) | 178 (93.7) |  |
| Other invasive carcinoma | 22 (4.9) | 12 (6.3) |  |
| Histologic tumor grade |  |  | 0.638 |
| Grade II | 74 (17.3) | 34 (18.9) |  |
| Grade III | 354 (82.7) | 146 (81.1) |  |
| Axillary tumor load |  |  | 0.463 |
| No positive lymph nodes | 288 (64.6) | 124 (65.3) |  |
| 1–3 positive lymph nodes | 104 (23.3) | 49 (25.8) |  |
| ≥4 positive lymph nodes | 54 (12.1) | 17 (8.9) |  |
| Lymphovascular invasion |  |  | 0.061 |
| Absent | 290 (65) | 138 (72.6) |  |
| Present | 156 (35) | 52 (27.4) |  |
| Adjuvant chemotherapy |  |  | 0.553 |
| Yes | 421 (94.4） | 175 (92.1） |  |
| No | 10 (2.2） | 6 (3.2） |  |
| Unknown | 15 (3.4) | 9 (4.7) |  |
| Adjuvant radiotherapy |  |  | 0.682 |
| Yes | 155 (34.7) | 63 (33.2) |  |
| No | 276 (61.9) | 118 (62.1) |  |
| Unknown | 15 (3.4) | 9 (4.7) |  |

BMI: Body Mass Index; SLNB: Sentinel Lymph Node Biopsy;

| **Table S2 Comparison of sonographic features in training and validation sets** | | | |
| --- | --- | --- | --- |
| **Characteristics** | **Training set (n=446)** | **Validation set (n=190)** | **P value** |
| Tumor size in ultrasound (mm) | 24.3 ± 9.1 | 24.1 ± 8.8 | 0.752 |
| Orientation |  |  | 0.706 |
| Parallel | 392 (87.9) | 169 (88.9) |  |
| Vertical | 54 (12.1) | 21 (11.1) |  |
| Shape |  |  | 0.728 |
| Regular | 73 (16.4) | 29 (15.3) |  |
| Irregular | 373 (83.6) | 161 (84.7) |  |
| Margin |  |  | 0.749 |
| Circumscribed | 60 (13.5) | 29 (15.3) |  |
| Angular/spiculated | 169 (37.9) | 67 (35.2) |  |
| Indistinct microlobular | 217 (48.6) | 94 (49.5) |  |
| Echogenic halo |  |  | 0.6 |
| Absent | 431 (96.6) | 182 (95.8) |  |
| Present | 15 (3.4) | 8 (4.2) |  |
| Echo pattern |  |  | 0.683 |
| Hypoechoic | 237 (53.1) | 99 (52.1) |  |
| Mixed hypo/iso-echoic | 178 (39.9) | 74 (38.9) |  |
| Mixed hypo/an-echoic | 31 (7.0) | 17 (9.0) |  |
| Posterior acoustic pattern |  |  | 0.37 |
| Shadow | 56 (12.6) | 23 (12.1) |  |
| Enhancement | 171 (38.3) | 84 (44.2) |  |
| Mixed change | 219 (49.1) | 83 (43.7) |  |
| Calcification |  |  | 0.629 |
| Absent | 339 (76.0) | 141 (74.2) |  |
| Present | 107 (24.0) | 49 (25.8) |  |
| Suspicious lymph nodes in ultrasound | |  | 0.839 |
| Absent | 337 (75.6) | 145 (76.3) |  |
| Present | 109 (24.4) | 45 (23.7) |  |
| Number of malignant features |  |  | 0.782 |
| No malignant feature | 49 (11.0) | 24 (12.6) |  |
| 1-2 malignant features | 293 (65.7) | 125 (65.8) |  |
| 3 or more malignant features | 104 (23.3) | 41 (21.6) |  |

| **Table S3 Comparison of mammographic features in training and validation sets** | | | |
| --- | --- | --- | --- |
| **Characteristics** | **Training set (n=316)** | **Validation set (n=142)** | **P value** |
| Breast density |  |  | 0.368 |
| Not dense | 100 (31.6) | 39 (27.5) |  |
| Dense | 216 (68.4) | 103 (72.5) |  |
| Lesion type |  |  | 0.985 |
| Mass | 227 (71.8) | 102 (71.9) |  |
| Calcification only | 7 (2.2) | 4 (2.8) |  |
| Architectural distortion | 10 (3.2) | 4 (2.8) |  |
| Asymmetry | 65 (20.6) | 28 (19.7) |  |
| Normal mammographic findings | 7 (2.2) | 4 (2.8) |  |
| Calcification |  |  | 0.666 |
| Absent | 220 (69.6) | 96 (67.6) |  |
| Present | 96 (30.4) | 46 (32.4) |  |
| Mass shape |  |  | 0.034 |
| Oval/round | 57 (18.0) | 40 (28.2) |  |
| Irregular | 170 (53.8) | 62 (43.6) |  |
| None | 89 (28.2) | 40 (28.2) |  |
| Mass margin |  |  | 0.289 |
| Circumscribed | 15 (4.7) | 12 (8.4) |  |
| Not circumscribed | 212 (67.1) | 90 (63.4) |  |
| None | 89 (28.2) | 40 (28.2) |  |
| Calcification morphology |  |  | 0.246 |
| Amorphous | 12 (3.8) | 2 (1.4) |  |
| Coarse heterogeneous | 5 (1.6) | 2 (1.4) |  |
| Fine pleomorphic | 53 (16.8) | 21 (14.8) |  |
| Fine branching | 4 (1.3) | 2 (1.4) |  |
| Benign | 22 (6.9) | 19 (13.4) |  |
| None | 220 (69.6) | 96 (67.6) |  |
| Calcification distribution |  |  | 0.873 |
| Regional | 7 (2.2) | 2 (1.4) |  |
| Grouped | 58 (18.4) | 27 (19.0) |  |
| Segmental | 9 (2.8) | 4 (2.8) |  |
| Branching | 4 (1.3) | 1 (0.7) |  |
| Scattered | 18 (5.7) | 12 (8.5) |  |
| None | 220 (69.6) | 96 (67.6) |  |
| Number of malignant features |  |  | 0.178 |
| No malignant feature | 67 (21.2) | 27 (19.0) |  |
| 1-2 malignant features | 32 (10.1) | 23 (16.2) |  |
| 3 or more malignant features | 217 (68.7) | 92 (64.8) |  |

| **Table S4 The recurrence percentages for each feature** | | | | | | | | |
| --- | --- | --- | --- | --- | --- | --- | --- | --- |
| **Clinicopathological characteristics** | | | **Sonographic features** | | | **Mammographic features** | | |
| **Variables** | **Training set (n=446)** | **Validation set (n=190)** | **Variables** | **Training set (n=446)** | **Validation set (n=190)** | **Variables** | **Training set (n=316)** | **Validation set (n=142)** |
| Age (year) |  |  | Orientation |  |  | Breast density |  |  |
| ＜55 | 39 (8.7) | 11 (5.8) | Parallel | 59 (13.2) | 27 (14.2) | Nondense | 16 (5.1) | 9 (6.3) |
| ≥55 | 31 (7.0) | 19 (10.0) | Vertical | 11 (2.5) | 3 (2.1) | Dense | 27 (8.5) | 13 (9.2) |
| Menopausal status |  |  | Shape |  |  | Lesion type |  |  |
| Premenopause | 30 (6.7) | 8 (4.2) | Regular | 10 (2.2) | 7 (3.7) | Mass | 32 (10.1) | 14 (9.9) |
| Menopause | 40 (9.0) | 22 (11.6) | Irregular | 60 (13.5) | 23 (12.1) | Calcification only | 0 (0.0) | 1 (0.7) |
| Tumor size in pathology (mm) |  |  | Margin |  |  | Architectural distortion | 1 (0.3) | 0 (0.0) |
| ≤20 | 21 (4.7) | 13 (6.8) | Circumscribed | 5 (1.1) | 4 (2.1) | Asymmetry | 9 (2.9) | 7 (4.9) |
| ＞20 | 49 (11.0) | 17 (8.9) | Angular/spiculated | 40 (9.0) | 14 (7.4) | Normal mammographic findings | 1 (0.3) | 0 (0.0) |
| Type of surgery |  |  | Indistinct/microlobular | 25 (5.6) | 12 (6.3) | Calcification |  |  |
| Breast-conserving surgery | 12 (2.7) | 3 (1.6) | Echogenic halo |  |  | Absent | 26 (8.2) | 14 (9.9) |
| Mastectomy | 58 (13.0) | 27 (14.2) | Absent | 67 (15.0) | 29 (12.1) | Present | 17 (5.4) | 8 (5.6) |
| Axillary surgery |  |  | Present | 3 (0.7) | 1 (0.5) | Mass shape |  |  |
| SLNB | 19 (4.3) | 9 (4.7) | Echo pattern |  |  | Oval/round | 7 (2.2) | 7 (4.9) |
| Axillary dissection | 51 (11.4) | 21 (11.1) | Hypoechoic | 31 (7.0) | 17 (8.9) | Irregular | 25 (7.9) | 7 (4.9) |
| Histological type |  |  | Mixed hypo/iso-echoic | 35 (7.8) | 12 (6.3) | None | 11 (3.4) | 8 (5.6) |
| Invasive ductal carcinoma | 66 (14.8) | 29 (15.3) | Mixed hypo/an-echoic | 4 (0.9) | 1 (0.5) | Mass margin |  |  |
| Other invasive carcinoma | 4 (0.9) | 1 (0.5) | Posterior acoustic pattern |  |  | Circumscribed | 0 (0.0) | 2 (1.4) |
| Histological grade |  |  | Enhancement | 19 (4.3） | 16 (8.4） | Noncircumscribed | 32 (10.1) | 12 (8.5) |
| Grade II | 13 (2.9) | 6 (3.2) | Shadow | 21 (4.7) | 2 (1.1) | None | 11 (3.4) | 8 (5.6) |
| Grade III | 54 (12.1) | 23 (12.1) | Mixed change | 30 (6.7) | 12 (6.3) | Calcification morphology |  |  |
| Axillary lymph node load |  |  | Calcification |  |  | Amorphous | 4 (1.3) | 0 (0.0) |
| No positive lymph node | 34 (7.6) | 16 (8.4) | Absent | 50 (11.2) | 19 (10.0) | Coarse heterogeneous | 0 (0.0) | 0 (0.0) |
| 1–3 positive lymph nodes | 15 (3.4) | 5 (2.6) | Present | 20 (4.5) | 11 (5.8) | Fine pleomorphic | 8 (2.5) | 4 (2.8) |
| ≥4 positive lymph nodes | 21 (4.7) | 9 (4.7) | Suspicious lymph nodes in US |  |  | Fine branching | 1 (0.3) | 1 (0.7) |
| Lymphovascular invasion |  |  | Absent | 43 (9.6) | 23 (12.1) | Benign | 4 (1.3) | 3 (2.1) |
| Absent | 34 (7.6) | 16 (8.4) | Present | 27 (6.1) | 7 (3.7) | None | 26 (8.2) | 14 (9.9) |
| Present | 36 (8.1) | 14 (7.4) | Number of malignant features |  |  | Calcification distribution |  |  |
| Ki-67 |  |  | No malignant feature | 8 (1.8) | 5 (2.6) | Regional | 0 (0.0) | 1 (0.7) |
| ≤40% | 30 (6.7) | 8 (4.2) | 1-2 malignant features | 28 (6.3) | 17 (8.9) | Grouped | 9 (2.9) | 3 (2.1) |
| ＞40% | 40 (9.0) | 22 (11.6) | 3 or more malignant features | 34 (7.6) | 8 (4.2) | Segmental | 3 (0.9) | 1 (0.7) |
| Adjuvant chemotherapy |  |  |  |  |  | Branching | 1 (0.3) | 1 (0.7) |
| Yes | 62 (13.9) | 27 (14.2) |  |  |  | Scattered | 4 (1.3) | 2 (1.4) |
| No | 6 (1.3) | 3 (1.6) |  |  |  | None | 26 (8.2) | 14 (9.9) |
| Unknown | 2 (0.4) | 0 (2.8) |  |  |  | Number of malignant features |  |  |
| Adjuvant radiotherapy |  |  |  |  |  | No malignant features | 7 (2.2) | 2 (1.4) |
| Yes | 30 (6.7) | 10 (5.3) |  |  |  | 1-2 malignant features | 2 (0.6) | 1 (0.7) |
| No | 38 (8.5) | 20 (10.5) |  |  |  | 3 or more malignant features | 34 (10.8) | 19 (13.3) |
| Unknown | 2 (0.4) | 0 (0.0) |  |  |  |  |  |  |
| BMI |  |  |  |  |  |  |  |  |
| <25 | 52 (11.7) | 18 (9.5) |  |  |  |  |  |  |
| 25-30 | 18 (4.3) | 12 (6.3) |  |  |  |  |  |  |
| >30 | 0 (0.0) | 0 (0.0) |  |  |  |  |  |  |


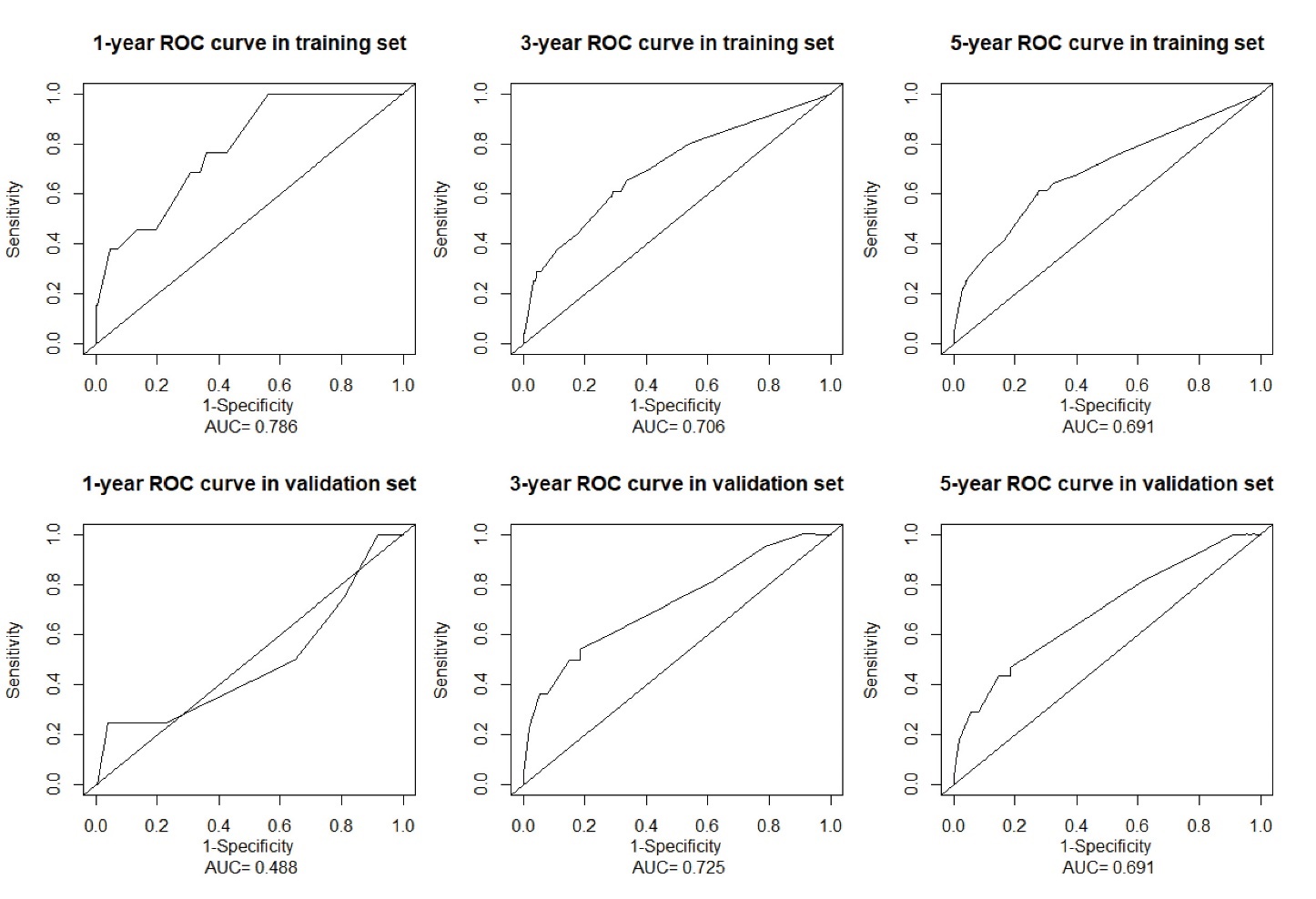


Figure S1 ROC curves of the established nomogram and the corresponding AUC.


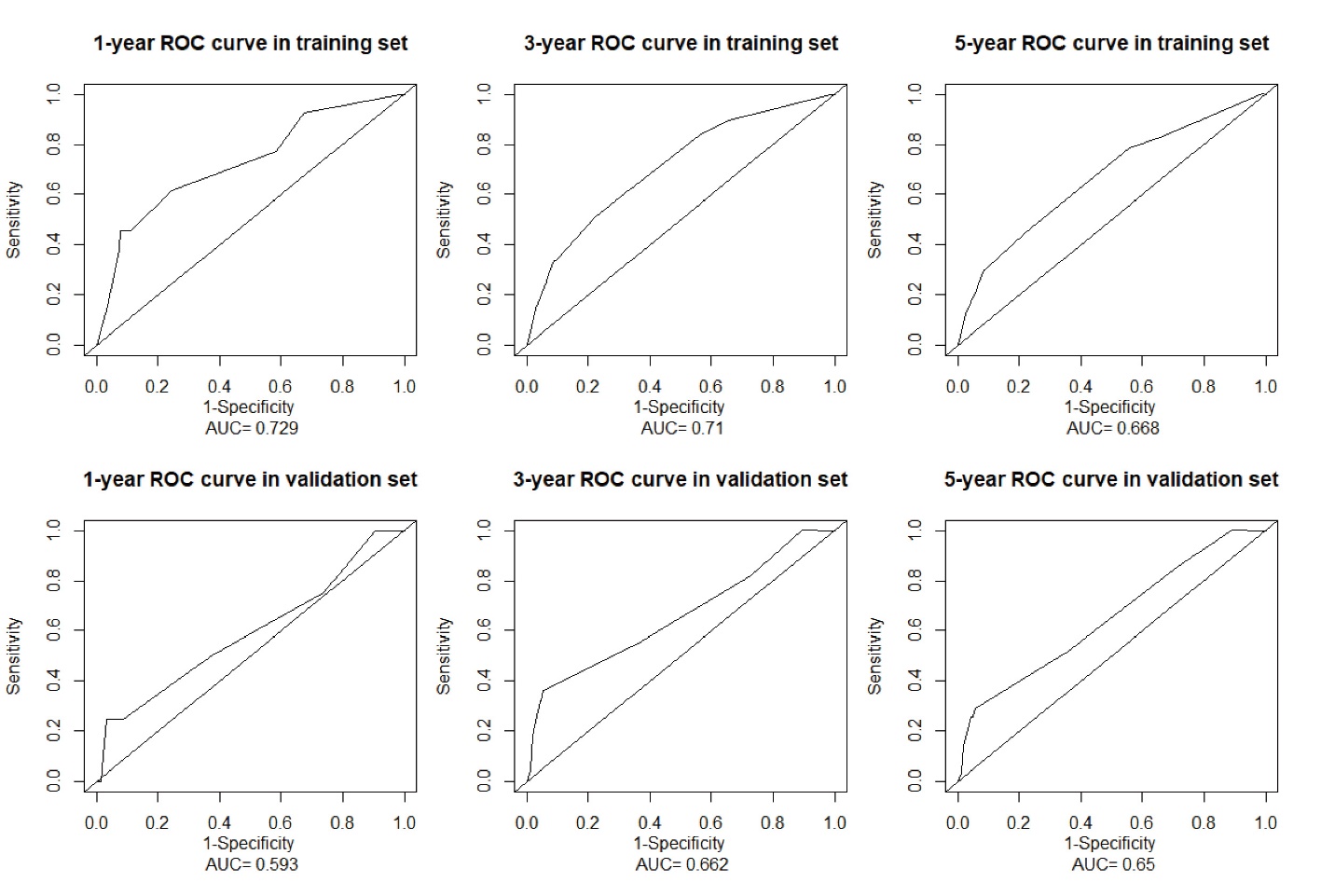


Figure S2 ROC curves of the AJCC staging system and the corresponding AUC.
